# Botulinum toxin treatment may improve myoelectric pattern recognition in robot-assisted stroke rehabilitation

**DOI:** 10.3389/fnins.2024.1364214

**Published:** 2024-02-29

**Authors:** Zhiyuan Lu, Yingchun Zhang, Sheng Li, Ping Zhou

**Affiliations:** ^1^School of Rehabilitation Science and Engineering, University of Health and Rehabilitation Sciences, Qingdao, China; ^2^Department of Biomedical Engineering, Desai Sethi Urology Institute, Miami Project to Cure Paralysis, University of Miami, Coral Gables, FL, United States; ^3^Department of Physical Medicine and Rehabilitation, University of Texas Health Science Center at Houston, Houston, TX, United States

**Keywords:** botulinum toxin, electromyography, myoelectric pattern recognition, robot-assisted rehabilitation, stroke

## 1 Introduction

Robot-assisted therapy is an effective treatment option for improving motor function in patients with neurological injury such as stroke, spinal cord injury, and cerebral palsy. Robot-assisted training facilitates improvements in motor performance necessary for completing activities of daily living. Active robotic training that takes the user's voluntary effort (or intent) into account can achieve better outcomes compared to passive training (Hu et al., 2009). Robots for active training are often driven by motion intents extracted from surface electromyography (EMG) signals. Compared with conventional proportional (Hu et al., 2009) or on-off (Hu et al., 2013) control strategies, myoelectric pattern recognition (Lu et al., 2017b) has the advantage of simultaneously controlling multiple degrees of freedom, an essential feature for increasing control of dexterity.

Unfortunately, despite the wide application of myoelectric pattern recognition in prosthesis control in amputees, relatively few have used it in patients with neurological injury, possibly because of the challenges associated with interference from spasticity. Spasticity and other types of muscle “overactivity” including spasms, clonus, and repetitive involuntary (spontaneous) motor unit activity associated with neurological injuries remain obstacles to robot-assisted therapy. For example, due to finger flexor spasticity and its associated involuntary activation, stroke survivors often flex their fingers during intended finger extension attempts (Kamper and Rymer, 2001). Among various treatment options, botulinum toxin therapy is often used and found to be effective at reducing spasticity. Although the relation between botulinum toxin treatment and motor function recovery is not clearly established (Ghasemi et al., 2013; Levy et al., 2019; Li et al., 2021), botulinum toxin therapy has demonstrated to be able to adequately suppress finger flexor spasticity and facilitate hand function in a subgroup of stroke survivors (Lee et al., 2018). Furthermore, the effectiveness of combining robot-assisted therapy and botulinum toxin treatment on motor function recovery has been reported (Gandolfi et al., 2019; Hung et al., 2021).

In the following sections, we discuss some of the confounding effects of spasticity (involuntary activity) and potential benefits of botulinum toxin treatment for facilitating myoelectric pattern recognition robot-assisted stroke rehabilitation.

## 2 Botulinum toxin treatment may improve voluntary muscle onset detection compromised by involuntary motor unit activity

After a stroke, involuntary motor unit activity is often observed at rest, particularly after a contraction, and may be interspersed with voluntary activity. It is technically challenging to selectively remove or reduce involuntary spikes using conventional signal processing methods because both involuntary and voluntary spikes have similar temporal and spatial characteristics. One common strategy for detection of voluntary muscle activity onset is based on amplitude measurements such as the root mean square or mean absolute value. A data instant with amplitude greater than a preset threshold is considered as the onset of muscle activity (Lu et al., 2017a,b). However, a resting data segment contaminated with involuntary discharges can be mistaken as active EMG and falsely trigger the robot. Although the chance of false triggering can be reduced by increasing the preset threshold, it may increase the rejection rate of voluntary motions, especially when there is severe muscle weakness.

Based on the observation that involuntary spikes sometimes have relatively stable firing rates and amplitude patterns (likely from the same motor units), several signal processing approaches have been proposed to overcome their influence on muscle onset detection (Zhang and Zhou, 2012; Liu et al., 2014a,b). These methods have not been tested in practical implementation of myoelectric control, due to some limitations. For example, sample entropy was reported to be able to detect muscle onset even if there are involuntary discharges (Zhang and Zhou, 2012). However, it is still unclear how to determine the global tolerance for the calculation of sample entropy in the case of real-time control. Therefore, muscle onset detection strategies applied in robot-assisted therapy are generally vulnerable to involuntary motor unit discharges, especially in patients with muscle weakness. Related to muscle onset detection, myoelectric pattern recognition is designed to extract motion intents from data segments that contain voluntary EMG signals. It is possible that involuntary motor unit discharges (either at rest or after the execution of a motion) are misclassified as voluntary motion intents. One strategy is to include the rest condition as a pattern in the candidate patterns, which are then recognized by the pattern recognition algorithm (Geng et al., 2013). Such a strategy can also be interfered because time domain (e.g., root mean square value) and frequency domain (e.g., mean and median power frequencies) features are sensitive to involuntary discharges.

Given the above, botulinum toxin treatment is expected to improve the performance of muscle onset detection due to its effectiveness in reducing involuntary muscle activity. Reliable muscle onset detection is essential for implementing myoelectric control.

## 3 Botulinum toxin treatment may improve classification performance

The myoelectric pattern recognition approach assumes that surface EMG features are consistent for a given muscle activation state associated with a particular task (motion intent) and different from one task to another. Surface EMG signals generated by the same motion intent in the presence or absence of spasticity may differ (i.e., increased time-variability or decreased stability of the EMG pattern). As a result, spasticity can degrade the performance of myoelectric pattern recognition. Our previous study suggests that EMG patterns extracted from post-stroke subjects are time-variant, and such time-variation compromises online myoelectric pattern recognition accuracy, whereas offline performance is less sensitive (Lu et al., 2019). Recognition accuracy was found to be less at low compared to moderate contraction strengths (Kopke et al., 2020), probably because the proportion of EMG power from involuntary discharges was higher at a low contraction strength. It is noteworthy that real-time myoelectric pattern recognition relies on EMG signals at the beginning of a motion intent (usually within 300 ms). During this period, the contraction level is relatively low and thus the performance of the muscle-machine interface is more likely to be affected by spasticity. This is consistent with our observation that the accuracy of real-time robot control (i.e., classification based on motion onset) was lower than the accuracy of offline recognition (i.e., classification throughout a motion) (Lu et al., 2019).

By reducing the muscle overactivity, botulinum toxin treatment is expected to facilitate myoelectric pattern recognition. In a study evaluating the effect of botulinum toxin injections on motor performance in chronic stroke subjects, it was found that both spasticity and muscle strength were reduced by the injections while motor performance of the weakened spastic muscle remained at similar levels before and after injections (Chen et al., 2020). Therefore, botulinum toxin treatment is promising to improve myoelectric pattern recognition performance for implementing real-time robotic control in stroke patients. It is likely that stroke patients with poor control of the robotic hand using myoelectric pattern recognition may achieve better control after botulinum toxin treatment.

## 4 Botulinum toxin treatment may improve range of motion

Some stroke patients have limited range of motion (ROM) on the affected side because of spasticity and contracture (Pandyan et al., 2003; Ro et al., 2020). Individual patients may have different combinations of spasticity and contracture (Lindberg et al., 2009). A longitudinal follow-up study of stroke patients using biomechanical measurements has suggested severe spasticity preceding contracture formation (Plantin et al., 2019). Attempts to stretch a patient's joint beyond the passive ROM may be resisted and painful. As a result, the robot ROM during therapy is usually set within the patient's passive ROM, although training with a larger ROM is potentially more beneficial. Depending on robot design, the ROM setting in a training task can be either preset (Lu et al., 2017b) or determined by the patient and the amount of assistance (Song et al., 2013). A patient may reach a larger ROM in both designs than through voluntary efforts (i.e., active ROM). Limb movements are primarily driven by the patient's voluntary muscle contraction within the active ROM (Feldman and Levin, 2016), whereas assistance becomes necessary or dominant beyond the active ROM. Botulinum toxin treatment (on its own or with other treatments) may increase both passive and active ROM (Marciniak et al., 2017; Lee et al., 2018; Picelli et al., 2019; Santamato, 2022; Trompetto et al., 2023), due in part to suppression of spasticity and associated involuntary activation of spastic muscles (Ro et al., 2020; Lindsay et al., 2021). It is possible to achieve the full ROM or at least enlarge the ROM of the robot in both passive and active training tasks. Therefore, botulinum toxin treatment may help release muscle from the restrictions of spasticity and contractures. This release should allow for more effective robotic training driven by myoelectric pattern recognition, leading to better recovery outcomes in stroke patients.

## 5 Summary

By reducing spasticity (overactivity), botulinum toxin treatment is expected to improve muscle onset detection for myoelectric control, as well as the performance of myoelectric pattern recognition for implementing real-time robotic control in stroke patients. Increased range of motion through botulinum toxin treatment may similarly create better conditions for enhanced myoelectric pattern recognition. These potential benefits indicate that combined botulinum toxin and myoelectric pattern recognition robotic training may be a promising stroke rehabilitation therapy.

## Author contributions

ZL: Conceptualization, Writing—original draft, Writing—review & editing, Funding acquisition. YZ: Conceptualization, Writing—review & editing. SL: Conceptualization, Writing—review & editing. PZ: Conceptualization, Funding acquisition, Writing—original draft, Writing—review & editing.
